# The Effect of Ocrelizumab on Anti‐JC Virus Antibody Index

**DOI:** 10.1002/brb3.71386

**Published:** 2026-05-14

**Authors:** Akash Virupakshaiah, Alexander J. Gill, Vinicius A. Schoeps, Emma Wilson, James Wilson, David Do, Amit Bar‐Or, Dina Jacobs, Joseph R. Berger

**Affiliations:** ^1^ Department of Neurology University of California San Francisco San Francisco California USA; ^2^ Department of Neurology, Penn Medicine Hospital of the University of Pennsylvania Philadelphia Pennsylvania USA; ^3^ Department of Neurology Johns Hopkins University School of Medicine Baltimore Maryland USA

**Keywords:** immunoglobulins, JC virus antibodies, multiple sclerosis, ocrelizumab, progressive multifocal leukoencephalopathy

## Abstract

**Purpose/Introduction:**

The anti‐JC virus (JCV) antibody index is used to stratify the risk of progressive multifocal leukoencephalopathy (PML) in multiple sclerosis (MS), particularly with natalizumab therapy. B‐cell‐depleting therapies may alter antibody levels and potentially confound the estimation of PML risk. This study evaluated the effect of ocrelizumab on anti‐JCV antibody indices during the first 2 years of treatment.

**Methods:**

We conducted a retrospective cohort study of 553 MS patients who initiated ocrelizumab between 2017 and 2019. Anti‐JCV antibody indices were measured using the STRATIFY JCV assay at baseline and approximately every 6 months prior to subsequent infusions. Linear mixed‐effects models were used to assess longitudinal changes in log‐transformed JCV indices, adjusting for age, sex, and ethnicity. Secondary analyses evaluated serum B‐cell counts and immunoglobulin levels. Sensitivity analyses addressed missing data.

**Results/Finding:**

There was no significant change in anti‐JCV antibody index over time following ocrelizumab initiation (mean percent change per infusion cycle −0.049%, 95% CI: −0.449 to 0.351; *p* = 0.81). Results were consistent across sensitivity analyses and did not differ by age or sex. In contrast, peripheral B‐cell counts declined significantly after treatment initiation and remained suppressed. Serum IgG, IgM, and IgA levels decreased modestly but significantly over time. JCV seroconversion occurred in 4.1% of initially seronegative patients, while seroreversion occurred in 6.6% of initially seropositive patients.

**Conclusion:**

Anti‐JCV antibody indices remain stable during the first 2 years of ocrelizumab treatment despite marked B‐cell depletion and modest reductions in serum immunoglobulins. These findings suggest that existing PML risk stratification tools based on anti‐JCV antibody indices remain applicable during early ocrelizumab therapy.

## Graphical abstract

Synopsis: This study of 553 MS patients found that anti‐JC virus (JCV) antibody indices remain stable during the first 2 years of ocrelizumab treatment. While peripheral B‐cells were significantly depleted and serum immunoglobulins (IgG, IgM, IgA) showed modest declines, the JCV antibody index did not change significantly. These results suggest that existing PML risk stratification tools based on JCV indices remain reliable during early ocrelizumab.

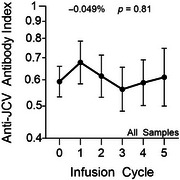

## 1 Introduction

The anti‐JC virus (JCV) antibody index predicts progressive multifocal leukoencephalopathy (PML) risk with natalizumab (Plavina et al. 2014). However, B‐cell‐depleting therapies may decrease anti‐JCV antibody levels, falsely lowering PML risk estimates (Plavina et al. 2014; Aladro et al. 2016; Alroughani et al. 2018; Ho et al. 2017). Rituximab has been shown to reduce anti‐JCV antibody indices (Bozic et al. 2011; Warnke et al. 2013) and causes hypogammaglobulinemia (Roberts et al. 2015; Barmettler et al. 2018). This study investigates the impact of ocrelizumab on anti‐JCV antibody indices over the first 2 years of treatment. Determining whether ocrelizumab reduces antibody indices would significantly influence PML risk stratification for patients on or transitioning from ocrelizumab.

## 2 Methods

### 2.1 Study Design

In this retrospective study, patient records from the MS Center at the University of Pennsylvania were systematically reviewed to identify individuals initiating ocrelizumab treatment between 2017 and 2019. Inclusion criteria encompassed a confirmed diagnosis of multiple sclerosis (MS) meeting the 2017 McDonald criteria, age 18 or older at the time of the initial ocrelizumab infusion, and the JCV antibody index was measured by STRATIFY JCV (Lee et al. 2013) in the clinical setting and repeated at approximately 6‐month intervals before the next scheduled ocrelizumab infusion. Ocrelizumab was administered to the patients at 600 mg every 6 months (Hauser et al. 2017). Patients undergoing alternative immunosuppressive therapies, including steroids exceeding 2 weeks, intravenous immunoglobulin (IVIG), and plasmapheresis, were excluded from the study to maintain homogeneity within the sample.

### 2.2 Data Collection

The Data Analytics Center at the University of Pennsylvania employed the Epic electronic medical record (EMR) and historical data uploaded to a secure portal to query patient data. Comprehensive manual reviews of externally uploaded JCV index results were conducted. B‐cell counts and immunoglobulin levels were also extracted.

### 2.3 Ethical Considerations and Statistical Analysis

This study was approved by the University of Pennsylvania's Institutional Review Board (IRB). The mean pre‐ocrelizumab JCV antibody index was compared to subsequent JCV antibody indices over time (per infusion cycle, log‐transformed) using mixed‐effects models adjusted for age, sex, and ethnicity. Differences in the rate of change by sex and age at treatment initiation were evaluated with the inclusion of interaction terms. Other immune markers (B‐cell count, IgG, IgM, and IgA) were also investigated using a similar analytical strategy, except for the use of a generalized mixed‐effects model set to a negative binomial family for B‐cell counts. Sensitivity analyses included: only subjects with paired JCV and B‐cell measurements, individuals with JCV measurements for all infusion cycles, and subjects with JCV indices measured in at least 70% of infusion cycles. For this last subgroup, a subsequent multiple imputation procedure (*n* = 20) assuming a multivariate normal distribution was performed to account for missing ethnicity and JCV indices. All data were analyzed using STATA 16.1 (College Station, TX), using two‐tailed tests and a *p* value < 0.05 as the criterion for statistical significance.

## 3 Results

### 3.1 Study Population

A total of 553 MS patients meeting the inclusion criteria were identified. Table 1 outlines the demographics of the cohort. A subset of 127 patients with serum B‐cell measurements mirrored the overall cohort's demographics. Tables S1 and S2 present details on the time elapsed between ocrelizumab infusion cycles, patient retention on treatment, and anti‐JCV index measurements.

**TABLE 1:** Descriptive table of baseline demographic characteristics of the study population.

	Full sample (*n *= 553)	Subset (*n *= 127)^a^
Sex, *n* (%)		
Male	161 (29.11)	43 (34.13)
Female	392 (70.89)	83 (65.87)
Age, years‐mean (SD)	44.38(11.99)	45.82 (12.15)
Ethnicity, *n* (%)		
White	372 (67.27)	88 (69.84)
Black	132 (23.87)	22 (17.46)
Hispanic White	22 (3.98)	6 (4.76)
Hispanic Black	7 (1.27)	3 (2.38)
Asian and Pacific Islander	3 (0.54)	—
Unknown	17 (3.07)	7 (5.56)
Follow‐up time, months, mean (SD)	25.86 (8.30)	27.42 (7.15)
Baseline immunoglobulin levels^b^		
IgG, mg/dL, median (IQR)	—	897 (858–1118)
IgA, mg/dL, median (IQR)	—	141 (115–238)
IgM, mg/dL, median (IQR)	—	105 (81–124)
Baseline B‐cell count, median (IQR)^c^	—	251.5 (111–421)

Abbreviations: SD; standard deviation; IQR; interquartile range.

^a^ Individuals with at least one B‐cell measurement.

^b^ 17 individuals with baseline measurement, 100 subjects have at least one measurement.

^c^ 86 individuals with baseline measurement.

### 3.2 Changes in Anti‐JCV Antibody Indices and Immunoglobulins Overtime

**FIGURE 1:**
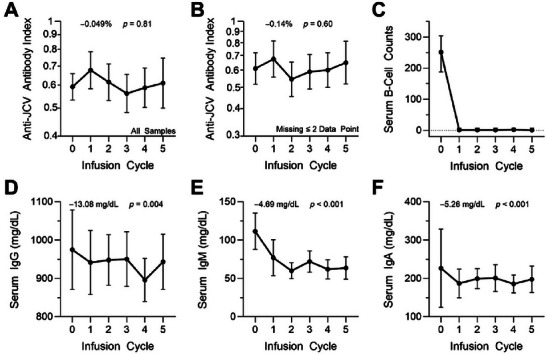
Ocrelizumab effects on JCV Antibody Index and Serum Immunoglobulins. Anti‐JCV antibody index at each ocrelizumab infusion cycle (A) in all MS patients (*n* = 536) and (B) in the subset with ≤ 2 missing data points (*n* = 215). All analyses of anti‐JCV data were performed on log‐transformed data. Data is presented on a logarithmic scale with means and 95% confidence intervals (CI). (C) Median B‐cell counts and 95% CI intervals at each ocrelizumab infusion cycle (approximately every 6 months; Cycle 1 encompasses the first two infusions). Mean and 95% CI of serum (D) IgG, (E) IgM, and (F) IgA levels at each ocrelizumab infusion cycle. Numbers above each graph show the mean change per infusion cycle and *p*‐values for change over the course of all cycles. Mean changes are presented as a percent change for anti‐JCV indices and as mg/dL change for immunoglobulins.

The mean difference in anti‐JCV antibody indices over time was not significant (−0.049% 95% CI: −0.449, 0.351, *p* value = 0.81) (Figure 1A). The sensitivity analysis provided similar findings (Figure 1B, and Table S3). No differential effect was observed by sex (*p* = 0.59) or age (*p* = 0.50). B‐cell counts dropped significantly after the initial ocrelizumab infusion (*p* < 0.001) and remained suppressed (Figure 1C). There was a significant decrease in IgG (*p* = 0.004), IgM (*p* < 0.001), and IgA levels (*p* < 0.001) over time (Table S4, and Figure 1D–F).

### 3.3 JCV Seroconversion and Seroreversion Analysis

Analysis of JCV serostatus changes revealed that 44.3% (*n* = 216) of subjects were seronegative at baseline. During the 2‐year follow‐up period, seroconversion occurred in 4.1% (*n* = 9) of initially seronegative patients, while seroreversion to negativity occurred in 6.6% (*n* = 18) of initially seropositive patients (*p *< 0.0001). Stratified analyses by baseline JCV serostatus identified statistically significant subgroup‐specific trends: a small decrease in the anti‐JCV antibody index among baseline seropositive patients and a small increase among baseline seronegative patients. In both groups, the estimated mean percent change per infusion cycle was less than 2% (Table S3 and Figure S1).

## 4 Discussion

In this retrospective cohort study, we found no significant change in anti‐JCV antibody indices in MS patients within the first 2 years of ocrelizumab treatment. While B‐cell counts significantly decreased, as expected with ocrelizumab, there were only modest reductions in IgG, IgM, and IgA serum levels, likely of limited clinical significance. This decline in immunoglobulin levels, a recognized effect of ocrelizumab,15 raises considerations about long‐term consequences of ocrelizumab and its effect on serum immunoglobulins, and potential effect on anti‐JCV antibodies. Given that ocrelizumab depletes CD20+ B‐cells and not plasma cells (CD20−), the stability of anti‐JCV antibody indices and only mild decreases in serum immunoglobulins in this study may be attributed to the persistence of active plasmablasts (Sabatino et al. 2019).

Our findings are consistent with a prior observational study that showed no significant change in anti‐JCV antibody indices during up to 24 months of ocrelizumab treatment (Rempe et al. 2020). In contrast, several prior studies, often involving smaller ocrelizumab‐treated cohorts or pooled anti‐CD20 populations, have reported modest reductions in anti‐JCV antibody indices. (Prezioso et al. 2021; Sgarlata et al. 2022; Baber et al. 2018). Baber et al. reported that rituximab treatment was associated with a significant reduction in JCV antibody indices in 76% of patients, with a mean decrease of 14% over 450 days, along with reduced immunoglobulin levels, particularly IgM (Baber et al. 2018). Similarly, Sgarlata et al. observed significant reductions in mean JCV index values in patients treated with rituximab or ocrelizumab (1.87 ± 1.54 vs. 1.57 ± 1.16, *p* = 0.003) and alemtuzumab (2.68 ± 1.15 vs. 1.50 ± 1.24, *p* = 0.005) (Sgarlata et al. 2022). Prezioso et al. also reported a statistically significant decrease in anti‐JCV index values over 1 year of ocrelizumab treatment (2.24 ± 1.53 vs. 1.56 ± 1.38, *p* < 0.05) (Prezioso et al. 2021). In contrast, our study demonstrates the stability of anti‐JCV antibody indices over 2 years of ocrelizumab treatment, which may be explained by several methodological differences, including a substantially larger cohort size (*n* = 553) providing greater statistical power compared with prior studies such as Baber et al. (*n* = 71) and Prezioso et al. (*n* = 42), a longer follow‐up duration relative to studies with shorter observation periods, and the avoidance of heterogeneous grouping of different anti‐CD20 agents, as rituximab and ocrelizumab differ in pharmacokinetics and depth of B‐cell depletion.

The development of PML is influenced by various risk factors, such as the presence of anti‐JCV antibodies, immunosuppressant class and duration, and recent prior use of certain immunosuppressants. Among patients receiving ocrelizumab, 16 cases of PML have been reported as of June 2024; 12 of 16 have been considered “carry‐over” patients as they had previously been on a disease‐modifying therapy with an established risk of PML (10 natalizumab, 1 fingolimod, 1 dimethyl fumarate) (Genentech 2023).

While this study has limitations, including its retrospective single‐center design and incomplete information about patients' previous disease‐modifying therapies, it also has several strengths that enhance the reliability of our findings. Our large patient cohort (*n* = 553) provides substantial statistical power, and the 2‐year follow‐up period captures both acute and sustained effects of ocrelizumab treatment. Despite missing data at later time points, our robust mixed‐effects modeling approach appropriately handles incomplete observations while providing valid estimates of the antibody trajectory.

These findings provide valuable insights into the short‐term impact of ocrelizumab on anti‐JCV antibody indices and suggest that current PML risk stratification tools may remain applicable during the first 2 years of treatment. However, longitudinal studies with extended follow‐up are needed to determine long‐term effects, and studies examining novel anti‐CD19 therapies that directly target plasma cells could provide additional insights into B‐cell versus plasma cell contributions to antibody maintenance. Until such data are available, cautious interpretation of anti‐JCV antibody results in MS patients on ocrelizumab therapy beyond 2 years remains prudent.

## Author Contributions

**Akash Virupakshaiah**: conceptualization, investigation, writing – original draft, methodology, validation, visualization, writing – review and editing, formal analysis, data curation. **Alexander J. Gill**: conceptualization, investigation, data curation, software, formal analysis, visualization, validation, methodology, writing – review and editing. **Vinicius A. Schoeps**: writing – review and editing, visualization, validation, methodology, software, formal analysis, supervision. **Emma Wilson**: data curation, methodology, writing – review and editing, validation, visualization. **James Wilson**: methodology, validation, visualization, writing – review and editing, data curation. **David Do**: methodology, supervision, writing – review and editing. **Amit Bar‐Or**: writing – review and editing, methodology, supervision, resources. **Dina Jacobs**: conceptualization, investigation, methodology, validation, writing – review and editing, supervision, resources, visualization. **Joseph R. Berger**: conceptualization, investigation, funding acquisition, methodology, validation, visualization, writing – review and editing, supervision, resources.

## Funding

This work was supported by a research grant from Roche/Genentech.

## Conflicts of Interest

Akash Virupakshaiah is supported by Fellowship Grants from Biogen, EMD Serono, and Novartis (2022–2024); and is a recipient of the National Multiple Sclerosis Society (NMSS) Sylvia Lawry Fellowship Award (Grant#FP‐2307‐41848, 2024–2025), and Young Investigator Award from the Race to Erase MS. Alexander J. Gill had received personal compensation as an employee of Novartis Pharmaceutical Corporation and has a non‐compensated role as President and Member of the Board of Directors of the Autoimmune Neurology Research Consortium, Inc. Amit Bar‐Or has received personal fees for advisory board participation and/or consulting from Abata, Accure, Atara, Biogen, Bristol Myers Squibb/Celgene/Receptos, GlaxoSmithKline, Gossamer, Horizon, Immunic, Janssen/Actelion, Medimmune, Merck/EMD Serono, Novartis, Roche/Genentech, Sangamo, Sanofi‐Genzyme, and Viracta; and has received grant support to the University of Pennsylvania from Biogen, Merck/EMD Serono, Novartis, and Roche/Genentech. Dina Jacobs has received personal fees for advisory board participation and/or consulting from Alexion, Biogen, BMS, Cycle Pharma, Horizon, Merck/EMD Serono, Novartis, Roche/Genentech, Sanofi‐Genzyme, TG Therapeutics, and grant support to the University of Pennsylvania from Biogen Idec, Roche/Genentech, Merck/EMD Serono and Novartis. Joseph R. Berger reports the following: consultancies for Celgene/BMS: Cycle Pharma; Dice Therapeutics; Genentech/Roche; Gilead; Janssen/J & J; Merck; Morphic; Novartis; Sandoz; Seagen; Takeda. Population Bio; and Excision Bio; and TG Therapeutics. He is on the DSMB of MAPI. He has also spoken on behalf of TG Therapeutics. The other authors declare no conflicts of interest.

## Supporting information



Table S1: Anti‐CD20 infusions over time.
Table S2: Anti‐JCV measurements in relation to subsequent infusion.
Table S3: Mean % difference of JCV titers over time adjusted by age, sex, and ethnicity (white vs non‐white), per infusion cycle.
Table S4: Mean change in immune markers adjusted by age, sex, and ethnicity (white vs non‐white), per infusion cycle.
Figure S1: Ocrelizumab effects on JCV antibody index stratified by baseline JCV serostatus.

## Data Availability

Anonymized data are available on reasonable request to the corresponding author, subject to approval from the University of Pennsylvania/Penn Medicine.

## References

[brb371386-bib-0001] Aladro, Y. , R. Terrero , M. Cerezo , et al. 2016. “Anti‐JC Virus Seroprevalence in a Spanish Multiple Sclerosis Cohort: JC Virus Seroprevalence in Spain.” Journal of the Neurological Sciences 365: 16–21. 10.1016/J.JNS.2016.03.050. 27206867

[brb371386-bib-0002] Alroughani, R. , S. Akhtar , S. Ahmed , and J. Al‐Hashel . 2018. “A Longitudinal Study of JC Virus Serostatus Stability Among Multiple Sclerosis Patients.” Multiple Sclerosis and Related Disorders 20: 132–135. 10.1016/J.MSARD.2018.01.016. 29414286

[brb371386-bib-0003] Baber, U. , A. Bouley , E. Egnor , and J. A. Sloane . 2018. “Anti‐JC Virus Antibody Index Changes in Rituximab‐Treated Multiple Sclerosis Patients.” Journal of Neurology 265, no. 10: 2342–2345. 10.1007/S00415-018-8996-3/TABLES/1. 30109480

[brb371386-bib-0004] Barmettler, S. , M. S. Ong , J. R. Farmer , H. Choi , and J. Walter . 2018. “Association of Immunoglobulin Levels, Infectious Risk, and Mortality With Rituximab and Hypogammaglobulinemia.” JAMA Network Open 1, no. 7: e184169. 10.1001/JAMANETWORKOPEN.2018.4169. 30646343 PMC6324375

[brb371386-bib-0005] Bozic, C. , S. Richman , T. Plavina , et al. 2011. “Anti‐John Cunnigham Virus Antibody Prevalence in Multiple Sclerosis Patients: Baseline Results of STRATIFY‐1.” Annals of Neurology 70, no. 5: 742–750. 10.1002/ANA.22606. 22162056

[brb371386-bib-0006] Genentech . 2023. “Ocrelizumab and PML.” Published August. https://www.ocrelizumabinfo.com/content/dam/gene/ocrelizumabinfo/pdfs/progressive‐multifocal‐leukoencephalopathy.pdf.

[brb371386-bib-0007] Hauser, S. L. , A. Bar‐Or , G. Comi , et al. 2017. “Ocrelizumab Versus Interferon Beta‐1a in Relapsing Multiple Sclerosis.” New England Journal of Medicine 376, no. 3: 221–234. 10.1056/NEJMOA1601277. 28002679

[brb371386-bib-0008] Ho, P. R. , H. Koendgen , N. Campbell , B. Haddock , S. Richman , and I. Chang . 2017. “Risk of Natalizumab‐Associated Progressive Multifocal Leukoencephalopathy in Patients With Multiple Sclerosis: A Retrospective Analysis of Data From Four Clinical Studies.” Lancet Neurology 16, no. 11: 925–933. 10.1016/S1474-4422(17)30282-X. 28969984

[brb371386-bib-0009] Lee, P. , T. Plavina , A. Castro , et al. 2013. “A Second‐Generation ELISA (STRATIFY JCVTM DxSelect) for Detection of JC Virus Antibodies in Human Serum and Plasma to Support Progressive Multifocal Leukoencephalopathy Risk Stratification.” Journal of Clinical Virology 57, no. 2: 141–146. 10.1016/J.JCV.2013.02.002. 23465394

[brb371386-bib-0010] Plavina, T. , M. Subramanyam , G. Bloomgren , et al. 2014. “Anti‐JC Virus Antibody Levels in Serum or Plasma Further Define Risk of Natalizumab‐Associated Progressive Multifocal Leukoencephalopathy.” Annals of Neurology 76, no. 6: 802–812. 10.1002/ANA.24286. 25273271 PMC4282070

[brb371386-bib-0011] Prezioso, C. , A. Grimaldi , D. Landi , et al. 2021. “Risk Assessment of Progressive Multifocal Leukoencephalopathy in Multiple Sclerosis Patients During 1 Year of Ocrelizumab Treatment.” Viruses 13, no. 9: 1684. 10.3390/V13091684. 34578264 PMC8473394

[brb371386-bib-0012] Rempe, T. , A. Carlson , A. Miravalle , and T. V. Gyang . 2020. “Anti‐JCV Antibody Index Does Not Change During Ocrelizumab‐Treatment.” Multiple Sclerosis Journal–Experimental, Translational and Clinical 6, no. 3: 2055217320960510. 10.1177/2055217320960510. 33029356 PMC7522834

[brb371386-bib-0013] Roberts, D. M. , R. B. Jones , R. M. Smith , et al. 2015. “Rituximab‐Associated Hypogammaglobulinemia: Incidence, Predictors and Outcomes in Patients With Multi‐System Autoimmune Disease.” Journal of Autoimmunity 57: 60–65. 10.1016/J.JAUT.2014.11.009. 25556904

[brb371386-bib-0014] Sabatino, J. J. , A. K. Pröbstel , and S. S. Zamvil . 2019. “B Cells in Autoimmune and Neurodegenerative Central Nervous System Diseases.” Nature Reviews Neuroscience 20, no. 12: 728–745. 10.1038/S41583-019-0233-2. 31712781

[brb371386-bib-0015] Sgarlata, E. , C. G. Chisari , S. Toscano , et al. 2022. “Changes in John Cunningham Virus Index in Multiple Sclerosis Patients Treated With Different Disease‐Modifying Therapies.” Current Neuropharmacology 20, no. 10: 1978. 10.2174/1570159x19666211111123202. 34766895 PMC9886813

[brb371386-bib-0016] Warnke, C. , R. Ramanujam , T. Plavina , et al. 2013. “Changes to Anti‐JCV Antibody Levels in a Swedish National MS Cohort.” Journal of Neurology, Neurosurgery, and Psychiatry 84, no. 11: 1199–1205. 10.1136/JNNP-2012-304332. 23463870 PMC3812878

