# Supplementary material for: The Effect of Ocrelizumab on Anti‐JC Virus Antibody Index
Source: Brain Behav. 2026 May 14;16(5):e71386. doi: 10.1002/brb3.71386 (PMC13175193; doi:10.1002/brb3.71386)
Supplement: Supplementary file 1 — Table S1: Anti‐CD20 infusions over time. Table S2: Anti‐JCV measurements in relation to subsequent infusion. Table S3: Mean % difference of JCV titers over time adjusted by age, sex, and ethnicity (white vs non‐white), per infusion cycle. Table S4: Mean change in immune markers adjusted by age, sex, and ethnicity (white vs non‐white), per infusion cycle. Figure S1: Ocrelizumab effects on JCV antibody index stratified by baseline JCV serostatus. [file BRB3-16-e71386-s001.docx]

| **Table S1**: Anti-CD20 infusions over time | | | | | |  | | |  |  |  |  |
| --- | --- | --- | --- | --- | --- | --- | --- | --- | --- | --- | --- | --- |
| Infusion | Mean time elapsed between infusions* | SD | Treatment discontinuation** | | |  | | |  |  |  |  |
| 1st and 2nd | 6.89 | 1.77 | 10 | | |  | | |  |  |  |  |
| 2nd and 3rd | 6.71 | 1.50 | 41 | | |  | | |  |  |  |  |
| 3rd and 4th | 6.61 | 1.51 | 88 | | |  | | |  |  |  |  |
| 4th and 5th | 6.72 | 1.48 | 173 | | |  | | |  |  |  |  |
| 5th and 6th | 6.51 | 0.93 | 324 | | |  | | |  |  |  |  |
| Abbreviations: SD: standard deviation | |  |  | | |  | | |  |  |  |  |
| * In months |  |  |  | | |  | | |  |  |  |  |
| ** Indicate the cumulative number of individuals who did not receive the subsequent infusions | | | | | |  | | |  |  |  |  |
|  |  |  |  | | |  | | |  |  |  |  |
|  |  |  |  | | |  | | |  |  |  |  |
| **Table S2**: Anti-JCV measurements in relation to subsequent infusion | | | | | |  | | |  |  |  |  |
| Infusion | Median time elapsed* | IQR | Missing measurements | | |  | | |  |  |  |  |
| 1st | 2.07** | 1.3 - 3.3 | 0 | | |  | | |  |  |  |  |
| 2nd | 1.63 | 0.2 - 3.0 | 280 | | |  | | |  |  |  |  |
| 3rd | 1.37 | 0.6 - 3.0 | 281 | | |  | | |  |  |  |  |
| 4th | 1.15*** | 0.6 - 3.2 | 313 | | |  | | |  |  |  |  |
| 5th | 1.20 | 0.4 - 2.9 | 362 | | |  | | |  |  |  |  |
| 6th | 1.0 | 0.3 - 2.6 | 433 | | |  | | |  |  |  |  |
| Abbreviations: IQR: inter quartile range | | | | | |  | | |  |  |  |  |
| * In months, before subsequent infusion | | |  | | |  | | |  |  |  |  |
| ** 24 patients collected JCV > 12 months prior to first infusion | | | | | |  | | |  |  |  |  |
| *** 1 patient collected blood sample 15 months after infusion. His subsequent infusion occurred 20 months after the prior | | | | | |  | | |  |  |  |  |
| **Table S3**: Mean % difference of JCV titers over time adjusted by age, sex, and ethnicity (white vs non-white), per infusion cycle | | | | | | | | | | | |  |
|  | | | | n | | | Mean % diff. (95% CI) | | | p-value | |  |
| Full sample* | | | | 536 | | | -0.049% (-0.449, 0.351) | | | 0.81 | |  |
| JCV-positive | | | | 297 | | | -1.379% (-1.796, -0.959) | | | p <0.001 | |  |
| JCV-negative | | | | 239 | | | 1.551% (0.873, 2.236) | | | p<0.001 | |  |
| Individuals with JCV measurements at all time points | | | | 150 | | | -0.050% (-0.777, 0.702) | | | 0.91 | |  |
| Individuals with B-cell measurement | | | | 119 | | | -0.070% (-0.767, 0.642) | | | 0.85 | |  |
| Individuals missing ≤2 JCV measurements | | | | 208 | | | -0.140% (-0.668, 0.381) | | | 0.60 | |  |
| Individuals missing ≤2 JCV measurements + Multiple imputation** | | | | 215 | | | -0.130% (-0.628, 0.381) | | | 0.62 | |  |
| Abbreviations: diff: difference, CI: confidence interval | | | |  | | |  | | |  | |  |
| *17 patients with missing ethnicity were dropped out of the model | | | |  | | |  | | |  | |  |
| **A multivariate normal distribution and 20 datasets were used to impute Anti-JCV titers and ethnicity | | | | | | | | | |  | |  |
| **Table S4**: Mean change in immune markers adjusted by age, sex, and ethnicity (white vs non-white), per infusion cycle | | | | | | | | | | | | |
|  | | | | | n | | | Mean difference (95% CI) | | | p-value | |
| IgG, mg/dL | | | | | 93 | | | -13.08 (-21.90 - -4.26) | | | **0.004** | |
| IgA, mg/dL | | | | |  |  |  | -4.69 (-6.79 - -2.60) | | | **<0.001** | |
| IgM, mg/dL | | | | |  |  |  | -5.26 (-6.57 - -3.95) | | | **<0.001** | |

**
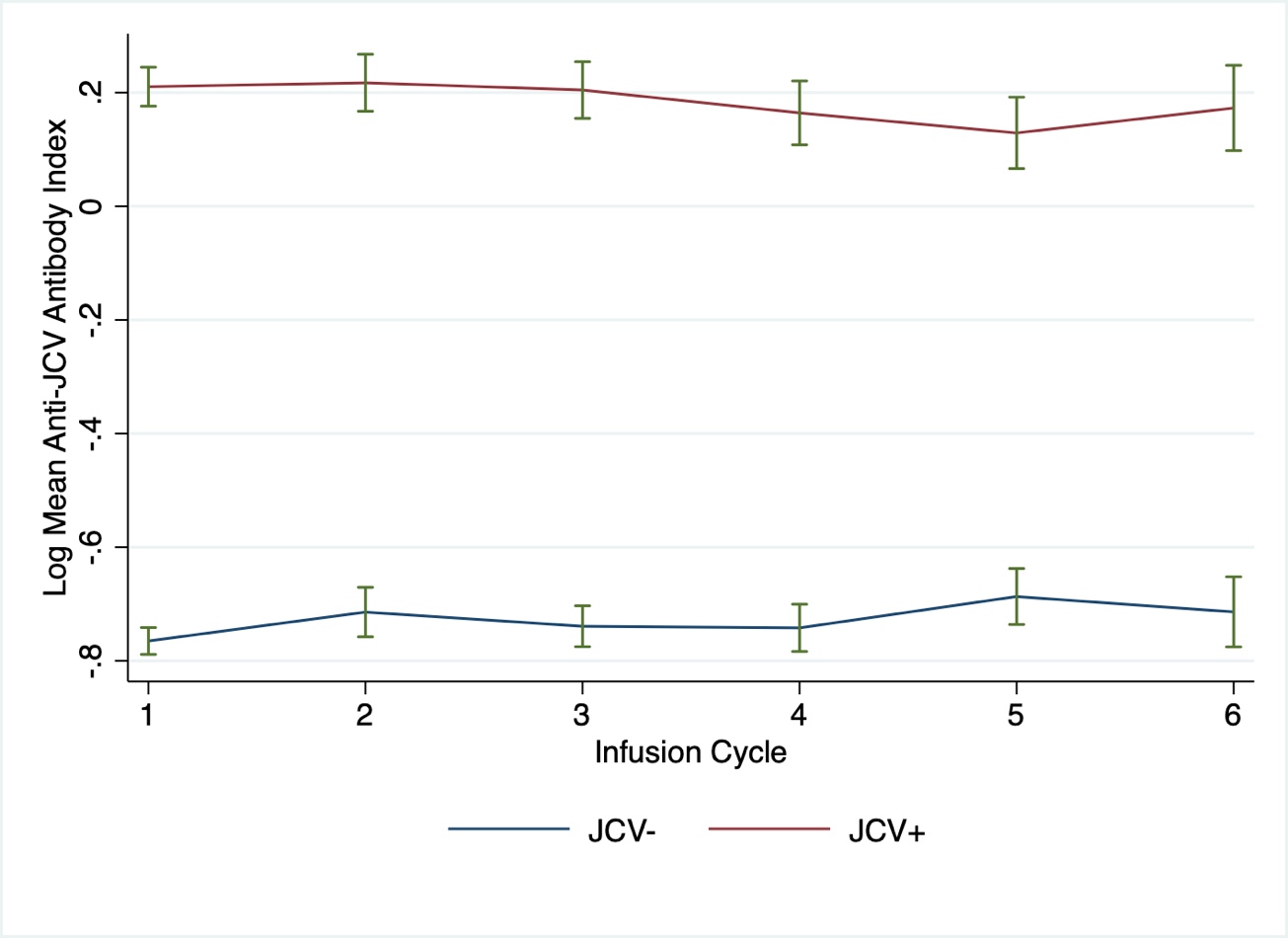
**

**Figure S1:** Ocrelizumab effects on JCV antibody index stratified by baseline JCV serostatus
